# Statistical-QoS Guaranteed Energy Efficiency Optimization for Energy Harvesting Wireless Sensor Networks

**DOI:** 10.3390/s17091933

**Published:** 2017-08-23

**Authors:** Ya Gao, Wenchi Cheng, Hailin Zhang

**Affiliations:** 1 State Key Laboratory of Integrated Services Networks, Xidian University, Xi’an 710071, China; wccheng@xidian.edu.cn (W.C.); hlzhang@xidian.edu.cn (H.Z.); 2 College of Physics and Electronic Information&Henan Key Laboratory of Electromagnetic Transformation and Detection, Luoyang Normal University, Luoyang 471934, China

**Keywords:** energy harvesting, quality of service (QoS), power control, energy efficiency optimization, wireless sensor networks

## Abstract

Energy harvesting, which offers a never-ending energy supply, has emerged as a prominent technology to prolong the lifetime and reduce costs for the battery-powered wireless sensor networks. However, how to improve the energy efficiency while guaranteeing the quality of service (QoS) for energy harvesting based wireless sensor networks is still an open problem. In this paper, we develop statistical delay-bounded QoS-driven power control policies to maximize the effective energy efficiency (EEE), which is defined as the spectrum efficiency under given specified QoS constraints per unit harvested energy, for energy harvesting based wireless sensor networks. For the battery-infinite wireless sensor networks, our developed QoS-driven power control policy converges to the Energy harvesting Water Filling (E-WF) scheme and the Energy harvesting Channel Inversion (E-CI) scheme under the very loose and stringent QoS constraints, respectively. For the battery-finite wireless sensor networks, our developed QoS-driven power control policy becomes the Truncated energy harvesting Water Filling (T-WF) scheme and the Truncated energy harvesting Channel Inversion (T-CI) scheme under the very loose and stringent QoS constraints, respectively. Furthermore, we evaluate the outage probabilities to theoretically analyze the performance of our developed QoS-driven power control policies. The obtained numerical results validate our analysis and show that our developed optimal power control policies can optimize the EEE over energy harvesting based wireless sensor networks.

## 1. Introduction

Energy harvesting offers a promising solution to prolong the lifetime of battery-powered wireless sensor networks. Different from the conventional energy supplies that suffer from limited lifetime, energy harvesting can provide the never-ending supply of energy for wireless sensor networks [1,2,3,4]. A large number of renewable energy sources, i.e., radio frequency (RF) signal, thermoelectric generator, vibration absorption device, etc. [5,6], can be exploited to harvest energy for wireless sensor nodes. Due to the random distribution and mobility of harvested energy powered sensor nodes, the energy harvesting often intermittently occurs, resulting in the very low energy efficiency for wireless sensor networks [7,8]. Therefore, it is very important to significantly increase the energy efficiency for energy harvesting based wireless sensor networks.

Recently, the energy efficiency in energy harvesting based wireless communications and networks were studied [9,10,11]. The authors of [9] developed the power allocation scheme to maximize the energy efficiency of orthogonal frequency division multiple access (OFDMA) based wireless powered communications. In order to improve energy efficiency, the authors of [10] jointly exploited full-duplex and massive multiple-input multiple-output (MIMO) technologies in energy harvesting based small cell networks. The joint energy allocation and energy cooperation scheme is proposed in [11] to maximize the energy efficiency for macro-small wireless networks, where each small-cell harvest energy from the energy sources. These works mainly optimize the energy efficiency under the short-term causal energy constraint. However, in practice, the harvested energy for the transmitter is sporadic and fluctuated [12]. The energy needs to be eventually accumulated up to a certain amount in the rechargeable battery for future data transmissions [8]. Hence, it is needed to resort to the long-term energy harvesting model [7,13,14,15,16]. In [7], a distributed power allocation, power splitting and relay selection algorithm is proposed to maximize the energy efficiency for cooperative clustered wireless sensor networks. The authors of [13,14] optimized the energy efficient resource allocation for the RF energy harvesting based collaborative mobile clouds. The authors of [15] maximized the cumulated throughput for RF based harvest-and-use and harvest-store-use schemes, respectively. Based on the Markov decision process framework, the net bit rates are maximized in [16]. However, how the battery capacity limitation impacts the resource allocation is not well studied in this literature. In fact, conservative or overly aggressive use of the harvested energy will either fail to utilize the excess energy or run out of the energy stored in the battery [3,4]. Even though some literature considered the battery-less energy harvesting sensor networks [17], the battery-less often needs the specific hardware design or the assumption for continuous energy supply. The energy harvesting based wireless sensor networks we considered are often intermittent and sporadic. Thus, it is imperative to optimize the energy efficiency under the long-term statistical energy constraint for battery-infinite and battery-finite energy harvesting based wireless networks, respectively.

Furthermore, we need to not only optimize energy efficiency alone, but also guarantee the QoS for energy harvesting based wireless sensor networks [18,19]. By integrating the information theory with statistical QoS provisioning principle, a great deal of effort has been made to maximize the effective capacity [20,21,22,23], which is defined as the maximum constant arrival rate that can be supported by the service rate under specified QoS requirements. However, only a few research works focus on QoS provisioning in energy harvesting based wireless sensor networks [24,25,26,27,28]. The magazine paper [24] reviewed the techniques that provide QoS guarantees for energy harvesting based wireless networks. The authors of [25,26] employed the deterministic QoS metric to evaluate the energy harvesting based wireless sensor networks. However, in 5G energy harvesting powered communications, statistical QoS guarantee, which is ignored in these works, is practical but more challenging. For statistical delay-bounded QoS provisioning, the authors of [27] maximized the effective capacity based on the state transition model while the authors of [28] analyzed the battery-outage and buffer-overflow probabilities to characterize the effective capacity of energy harvesting based wireless networks. However, due to the unreliable nature of energy harvesting, how the energy arrival rate affects the energy harvesting based wireless networks, which is not taken into account in both [27,28], is still an open problem.

To remedy the above deficiencies, in this paper, we propose the statistical delay-bounded QoS-driven power control policies to maximize the effective energy efficiency (EEE), which is defined as the achieved effective capacity per unit harvested energy, under the statistical average constraints and the battery capacity constraints for energy harvesting based wireless sensor networks. First, we convert the causality constraints into long-term statistical average constraints. Second, we formulate the EEE maximization problems for the battery-infinite and battery-finite energy harvesting based wireless sensor networks, respectively. Third, we develop the optimal power control policies to maximize the EEE of energy harvesting based wireless sensor networks. Our developed optimal power control policy in battery-infinite energy harvesting based wireless sensor networks varies from the Energy harvesting Water Filling (E-WF) scheme (under the very loose QoS requirement) to the Energy harvesting Channel Inversion (E-CI) scheme (under the very stringent QoS requirement), while our developed optimal power control policy in battery-finite energy harvesting based wireless sensor networks varies from the Truncated energy harvesting Water Filling scheme (T-WF) (under the very loose QoS requirement) to the Truncated energy harvesting Channel Inversion (T-CI) scheme (under the very stringent QoS requirement). For battery-finite energy harvesting based wireless sensor networks, we derive and analyze the statistical QoS-driven power control policies under the following three scenarios: (i) the average harvested energy constraint dominated optimal power control policy, (ii) the battery capacity constraint dominated optimal power control policy, and (iii) both the average harvested energy constraint and the battery capacity constraint dominated optimal power control policy. Furthermore, we analyze the outage probability for our developed optimal power control policy. The numerical obtained results validate our analyses and show that our proposed QoS-driven power control polices can maximize the EEE for energy harvesting based wireless sensor networks, thus enabling efficient and QoS-guaranteed energy harvesting wireless communications in wireless sensor networks.

The rest of this paper is organized as follows. Section 2 gives our QoS-guaranteed energy harvesting based wireless sensor network model and introduces the principle of effective energy efficiency. Section 3 and Section 4 develop the QoS-driven power control policies to maximize the effective energy efficiency for battery-infinite and battery-finite energy harvesting based wireless sensor networks, respectively. Section 5 analyzes the energy outage probabilities and the data-transmission outage probabilities. Section 6 numerically evaluates our developed QoS-driven power control polices for battery-infinite and battery-finite energy harvesting based wireless sensor networks, respectively. The paper concludes with Section 7.

## 2. System Model

**Figure 1:**
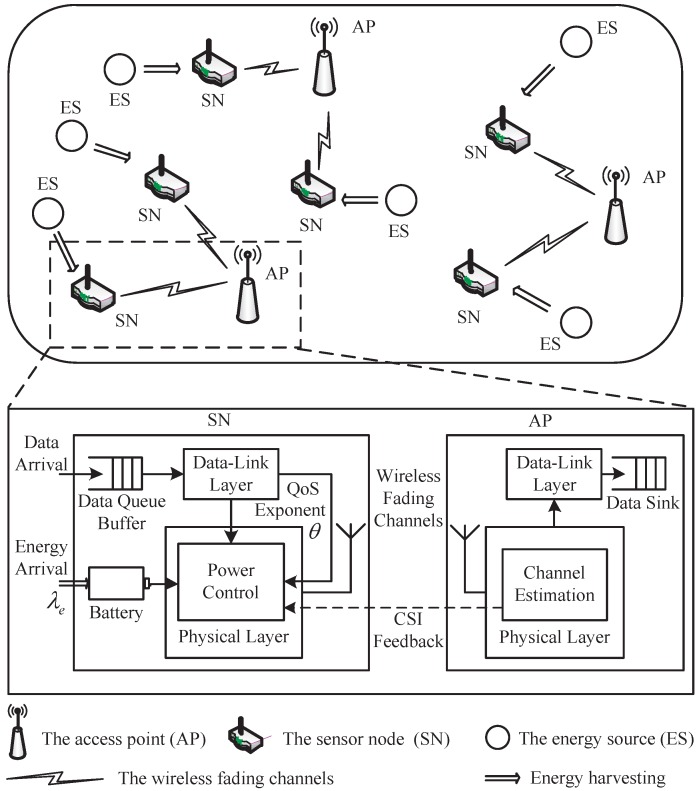
The system model for energy harvesting wireless sensor network.

We consider an energy harvesting based wireless sensor network model, as shown in Figure 1, where the energy harvesting enabled sensor nodes (SNs) communicate with the access point (AP). We concentrate on a discrete time system with a point-to-point link between the SN and WAP. Time division multiple access (TDMA) is employed for the SN-AP communications. In such scenario, incremental energy is harvested by the SN from the ambient energy sources and stored in the battery for data transmission.

A first-in-first-out (FIFO) data queue buffer is implemented at the SN, which contains the data packets from the upper-protocol-layer, as illustrated in Figure 1. The packets are divided into frames at the data-link layer and split into bit-streams at the physical layer. The channel state information (CSI) is estimated at the AP and reliably fed back to the SN. The SN needs to find the optimal power control policy based on the QoS constraint requested by the service, the CSI fed back from the AP, and the available energy harvested from the environments.

We denote by *B*, $E_{H}$ , and P[i] the total bandwidth of one SN-AP link, the average harvested energy, and the instantaneous transmit power, respectively, where *i* is the time index of the frame. The additive white Gaussian noise (AWGN) is denoted by $N_{0}$ . The channel power gains, denoted by g[i] , follow the stationary block fading channel model, where they keep unchanged within the time duration of one frame, but vary independently across different frames. The instantaneous channel signal-to-noise ratio (SNR), denoted by γ[i] , can be expressed as $\gamma \left[i\right]=E_{H}g\left[i\right]/N_{0}B$ . Moreover, we employ Nakagami-*m* fading channel model, which is very general and often best fits the land-mobile and indoor mobile multi-path propagations. The probability density function (PDF) of instantaneous channel SNR, denoted by $p_{\Gamma }\left(\gamma \right)$ , can be expressed as follows:

(1) $$p_{\Gamma }\left(\gamma \right)=\frac{\gamma^{m-1}}{\Gamma (m)}{\left(\frac{m}{\bar{\gamma }}\right)}^{m}\exp\left(-\frac{m}{\bar{\gamma }}\gamma \right),\gamma \geq 0,$$

where Γ(·) denotes the Gamma function, *m* represents the fading parameter of Nakagami-*m* distribution, and $\bar{\gamma }$ is the average received signal-to-noise ratio.

### 2.1. The Statistical Delay-Bounded QoS Guarantees

Based on large deviation principle (LDP), the author of [29] showed that, for a queueing system with stationary and ergodic arrival and service process, the queue length process Q(t)(t≥0) converges in distribution to a finite random variable Q(∞) that satisfies

(2) $$-\lim_{x\to \infty }\frac{\log\mathrm{Pr}(Q(\infty )\geq x)}{x}=\theta ,$$

which states that the probability of the queue length exceeding the queue length bound *x* decays exponentially as the bound *x* increases. The parameter θ ( θ>0 ), which is called *QoS exponent* [21], indicates the exponential decay rate dominated by the queue length bound. A large θ leads to a fast decay rate, which implies that a stringent QoS demand is supported. A small θ corresponds to a slow decay rate, which means that the system can provide a loose QoS requirement [30].

The sequence { R[i],i=1,2,… } is defined as a discrete-time stationary and ergodic stochastic service process, and $S\left[t\right]≜\sum_{i=1}^{t}R\left[i\right]$ is the partial sum of the service process over time sequence of i=1,2,…,t . The Gartner–Ellis limit of S[t] , expressed as $\Lambda_{C}\left(\theta \right)=\lim_{t\to \infty }\left(1/t\right)\log\left(E\left\{e^{-\theta S[t]}\right\}\right)$ , is a convex function differentiable for all real θ [29]. The instantaneous service rate R[i] can be derived as follows [31]:

(3) $$R\left[i\right]=T_{f}B\log_{2}\left(1+\mu \left[i\right]\gamma \left[i\right]\right),$$

where μ[i] is the power control policy. We define the power control policy as the proportion of transmit power in the average harvested energy. Thus, the instantaneous transmit power can be written as $P\left(\eta \left[i\right]\right)=\mu \left(\eta \left[i\right]\right)E_{H}.$ When the service rate sequence R[i] is stationary and time-uncorrelated, we can derive the effective capacity as follows [21]:

(4) $$C\left(\theta \right)≜-\frac{\Lambda_{C}\left(-\theta \right)}{\theta }=-\frac{1}{\theta }\log\left(E\left\{e^{-\theta R[i]}\right\}\right).$$

### 2.2. Effective Energy Efficiency in Energy Harvesting Based Wireless Sensor Networks

The SN harvests energy from the environments and stores it in the battery. The energy arrives at discrete time intervals with various amounts. We assume that the energy arrival process is stationary and ergodic, and thus can be modeled as the Poisson process with the arrival rate $\lambda_{e}$ [4,32]. Therefore, according to the Poisson process based energy arrival [4,32], the average harvested energy, denoted by $E_{H}$ , is equivalent to the energy arrival rate and can be derived as follows:

(5) $$E_{H}=E\left\{H[i]\right\}=\lambda_{e},$$

where H[i] is the harvested energy during the $i^{\mathrm{th}}$ time frame.

We aim to maximize the energy efficiency under the statistical delay-bounded QoS provisioning for energy harvesting based wireless sensor networks. Thus, we define the *effective energy efficiency* (EEE), denoted by $E_{e}$ , as the achieved effective capacity per unit harvested energy. Then, we can derive the EEE for energy harvesting based wireless sensor networks as follows:

(6) $$E_{e}≜\frac{C\left(\theta \right)}{E_{H}}.$$

Without loss of generality, we normalize the observation time interval. Thus, the terms of power and energy can be interchangeably used.

## 3. QoS-Driven Optimal Power Control Policy with Infinite Battery Capacity

In this section, we assume that the battery capacity is large enough to store the harvested energy without energy overflow. Conventionally, the power control schemes are functions of the instantaneous SNR γ[i] . However, for battery-infinite energy harvesting based wireless sensor networks, our QoS-driven power control policy, denoted by μ(η[i]) , needs to be adaptive to the instantaneous SNR γ[i] , the QoS exponent θ , and the energy arrival rate $\lambda_{e}$ . The variable $\eta \left[i\right]≜(\gamma \left[i\right],\theta ,\lambda_{e})$ is defined as the *QoS and energy based state information* (QSI).

### 3.1. Average Harvested Energy Constraint

We assume that the harvested energy is only used for transmission, i.e., energy required for processing is not taken into account [3,4]. Then, the instantaneous transmit power in energy harvesting based wireless sensor networks cannot exceed the available harvested energy, which can be formulated as follows:

(7) $$\sum_{i=1}^{t}P\left(\eta \left[i\right]\right)\leq \sum_{i=0}^{t-1}H\left[i\right],\;\forall t,$$

where P(η[i]) is the transmit power during the ith frame and the symbol notation H[0] denotes the amount of energy available in the battery at the initial time. The right-hand of Equation (7) is the summation of harvested energy from the initial time to (t−1)th frame because the harvested energy in the tth frame cannot be used for transmission at the same time. Since the discrete-time channel and the energy arrival process are both stationary and ergodic, the time average is equal to the statistical average for the harvested energy [33], which is shown as follows:

(8) $$\begin{array}{c} \left\{\begin{array}{c} \frac{1}{t}\sum_{i=1}^{t}P\left(\eta \left[i\right]\right)=\lim_{t\to \infty }\frac{1}{t}\sum_{i=1}^{t}P\left(\eta \left[i\right]\right)=E_{\gamma }\left\{P\left(\eta \left[i\right]\right)\right\};\\ \frac{1}{t-1}\sum_{i=0}^{t-1}H\left[i\right]=\lim_{t\to \infty }\frac{1}{t-1}\sum_{i=0}^{t-1}H\left[i\right]=E_{H}. \end{array}\right. \end{array}$$

In the following, we omit the time-index *i* for simplicity. When *t* is large enough, we substitute Equation (8) into Equation (7) and rewrite Equation (7) as follows:

(9) $$E_{\gamma }\left[P\left(\eta \right)\right]\leq E_{H},$$

which shows that the power control policy is constrained by the average harvested energy.

### 3.2. The Effective Energy Efficiency Maximization for Battery-Infinite Energy Harvesting Based Wireless Sensor Networks

We formulate the energy efficient optimization problem, denoted by P1 , to maximize EEE in battery-infinite energy harvesting based wireless sensor networks as follows by using Equations (4) and (6):

(10) $$P1:\arg\max_{\mu (\eta )}\left\{\frac{-\frac{1}{\theta }\log\int_{0}^{\infty }e^{-\theta T_{f}B\log_{2}\left(1+\mu \left(\eta \right)\gamma \right)}p_{\Gamma }\left(\gamma \right)d\gamma }{E_{H}}\right\},$$

subject to Equation (9) and μ(η)≥0 .

Since log(·) is a monotonically increasing function, the numerator of objective function in problem P1 can be simplified as follows:

(11) $$-\frac{1}{\theta }\log\int_{0}^{\infty }e^{-\theta T_{f}B\log_{2}\left(1+\mu \left(\eta \right)\gamma \right)}p_{\Gamma }\left(\gamma \right)d\gamma =-\frac{1}{\theta }\log\int_{0}^{\infty }{\left(1+\mu \left(\eta \right)\gamma \right)}^{-\theta T_{f}B}p_{\Gamma }\left(\gamma \right)d\gamma .$$

Due to the monotonicity of log(·) function and linearity of (1+μ(η)) , the numerator of objective function in problem P1 is strictly concave with respect to μ(η) . However, the problem P1 is still a non-convex optimization problem because of the variable in the denominator. In order to convert the problem P1 into a convex optimization problem, we assume the energy arrival rate $\lambda_{e}$ to be fixed for the energy harvesting based wireless sensor network. This kind of assumption is practical because the energy sources for the energy harvesting based wireless sensor networks are relatively stable during the short period and variable across the whole energy harvesting process. Therefore, we can solve problem P1 with fixed $\lambda_{e}$ and the solution of problem P1 is adopted to the energy harvesting based wireless networks with different values of $\lambda_{e}$ . Since log(·) is a monotonically increasing function, problem P1 can be simplified as the new problem P2 , which is formulated as follows:

(12) $$P2:\;\;\arg\min_{\mu (\eta )}\left\{\int_{0}^{\infty }{\left(1+\mu \left(\eta \right)\gamma \right)}^{-\beta }p_{\Gamma }\left(\gamma \right)d\gamma \right\},$$

subject to Equation (9) and μ(η)≥0 . The term $\beta =(\theta T_{f}B)/\log2$ is defined as the normalized QoS exponent. It is clear that the objective function of P2 is strictly convex and the item $E_{\gamma }\left(P\left(\eta \right)\right)$ in Equation (9) is linear with respect to μ(η) . Thus, problem P2 is a strictly convex optimization problem and the optimal solution for problem P2 is given by the following Theorem 1.

**Theorem** **1.** The optimal power control policy for the battery-infinite energy harvesting based wireless sensor networks, denoted by $\mu^{*}\left(\eta \right)$ , which is the solution of problem P2 , is determined by

(13) $$\begin{array}{c} \mu^{*}\left(\eta \right)=\left\{\begin{array}{cc} \frac{\lambda_{e}^{-\frac{1}{\beta +1}}}{\gamma_{\mathrm{in}}^{\frac{1}{\beta +1}}\gamma^{\frac{\beta }{\beta +1}}}-\frac{1}{\gamma }, & \gamma \geq \gamma_{\mathrm{in}};\\ 0, & \gamma <\gamma_{\mathrm{in}}, \end{array}\right. \end{array}$$

where $\gamma_{\mathrm{in}}$ is defined as the cut-off SNR threshold in the battery-infinite energy harvesting based wireless sensor networks and can be numerically obtained by substituting $\mu^{*}\left(\eta \right)$ into the following constraint:

(14) $$\int_{\gamma_{\mathrm{in}}}^{\infty }\mu^{*}\left(\eta \right)p_{\Gamma }\left(\gamma \right)d\gamma =1.$$

**Proof.**  The Lagrangian function of problem P2 is formulated as follows:

(15) $$L=\int_{0}^{\infty }{\left(1+\mu \left(\eta \right)\gamma \right)}^{-\beta }p_{\Gamma }\left(\gamma \right)d\gamma +\kappa \left(E_{\gamma }\left[P\left(\eta \right)\right]-\lambda_{e}\right),$$

where κ is the Lagrange multiplier. Then, the Karush–Kuhn–Tucker (KKT) conditions of problem P2 can be written as follows [34]:

(16) $$\left\{\begin{array}{c} -\beta \gamma {\left(1+\mu \left(\eta \right)\gamma \right)}^{-\beta -1}p_{\Gamma }\left(\gamma \right)+\kappa \lambda_{e}p_{\Gamma }\left(\gamma \right)=0;\\ \kappa \left(E_{\gamma }\left[P\left(\eta \right)\right]-\lambda_{e}\right)=0;\\ \kappa \geq 0. \end{array}\right.$$

Defining $\gamma_{\mathrm{in}}≜\kappa /\beta$ and solving Equation (16), we can obtain the optimal power control policy as shown in Equation (13), where $\gamma_{\mathrm{in}}$ can be numerically obtained from Equation (14). ☐

**Figure 2:**
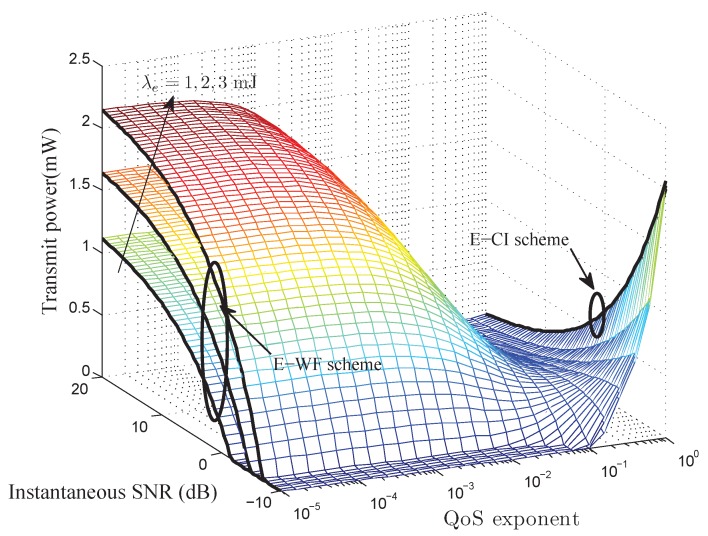
The QoS-driven power control policy for battery-infinite energy harvesting based wireless sensor networks with m=2 and $\bar{\gamma }=5\;\mathrm{dB}$ .

Theorem 1 gives the QoS-driven power control policy for battery-infinite energy harvesting based wireless sensor networks. To better understand the insights of Theorem 1, we plot the instantaneous transmit power control policy in Figure 2. Observing Figure 2, we have: (i) given energy arrival rate, when QoS exponent is very small, more power is assigned to the better channel and less power to the worse channel. However, when QoS exponent is very large, more power is assigned to the worse channel and less power to the better channel. (ii) The allocated power increases as the energy arrival rate increases. In addition, we can observe that the cut-off SNR threshold depends on $\lambda_{e}$ . Furthermore, we discuss two specific cases of Theorem 1 in following Remarks 1 and 2, which are the optimal power control policies under the very loose QoS constraint and the very stringent QoS constraint, respectively, for battery-infinite energy harvesting based wireless sensor networks.

**Remark** **1.** Under the very loose QoS constraint ( θ→0 ), the optimal energy harvesting power control policy for $\mu^{*}\left(\eta \right)$ converges to

(17) $$\begin{array}{c} \lim_{\theta \to 0}\mu^{*}\left(\eta \right)=\left\{\begin{array}{cc} \frac{1}{\lambda_{e}\gamma_{\mathrm{in}}}-\frac{1}{\gamma },\;\;\;\; & \gamma \geq \gamma_{\mathrm{in}};\\ 0, & \gamma <\gamma_{\mathrm{in}}, \end{array}\right. \end{array}$$

*which is referred to the Energy harvesting Water-Filling (E-WF) scheme. When the QoS constraint is very loose, our developed optimal power control policy converges to the E-WF scheme, where the water levels are dominated by the energy arrival rate and cut-off SNR threshold. The conventional staircase water-filling scheme [3] is the special case (θ=0) of the E-WF scheme.*

**Remark** **2.** Under the very stringent QoS constraint ( θ→∞ ), the optimal power control policy for energy harvesting based wireless networks $\mu^{*}\left(\eta \right)$ converges to

(18) $$\lim_{\theta \to \infty }\mu^{*}\left(\eta \right)=\frac{\sigma_{\mathrm{in}}}{\gamma },$$

*where $\sigma_{\mathrm{in}}=\lim_{\theta \to \infty }\left[{\left(\lambda_{e}\gamma_{\mathrm{in}}\right)}^{-\frac{1}{\beta +1}}-1\right]$ . We call the power control policy specified in Equation (18) the Energy harvesting Channel Inversion (E-CI) scheme.*

As illustrated in Figure 2, when θ varies from 0 to ∞, reflecting different delay-bounded QoS constraints, our developed QoS-driven energy harvesting power control policy swings between the E-WF scheme and the E-CI scheme. Using our developed optimal power control policy for battery-infinite energy harvesting based wireless sensor networks, we can derive the maximum EEE, denoted by $E_{e}^{*}\left(\theta ,\lambda_{e}\right)$ , as follows:

(19) $$\begin{array}{c} E_{e}^{*}\left(\theta ,\lambda_{e}\right)=-\frac{1}{\theta \lambda_{e}}\left\{\log\left[\gamma \left(m,\frac{m}{\bar{\gamma }}\gamma_{\mathrm{in}}\right)+{\left[\frac{\lambda_{e}m\gamma_{\mathrm{in}}}{\bar{\gamma }}\right]}^{\frac{\beta }{\beta +1}}\Gamma \left(m-\frac{\beta }{\beta +1},\frac{m\gamma_{\mathrm{in}}}{\bar{\gamma }}\right)\right]-\log\left[\Gamma (m)\right]\right\}, \end{array}$$

where γ(·,·) and Γ(·,·) denote the lower and upper incomplete Gamma functions, respectively.

## 4. QoS-Driven Optimal Power Control Policy with Finite Battery Capacity

In this section, we aim to maximize the EEE of energy harvesting based wireless sensor networks with finite battery capacity. Let $\tilde{\mu }\left(\eta \left[i\right]\right)$ denote by the QoS-driven power control policy in the ith frame and $\tilde{P}\left(\eta \left[i\right]\right)=\tilde{\mu }\left(\eta \left[i\right]\right)E_{H}$ denote by the transmit power in the ith frame for the SNs with finite battery capacity.

### 4.1. The Effective Energy Efficiency Maximization for Battery-Finite Energy Harvesting Based Wireless Sensor Networks

We denote by $B_{\max}$ the maximum battery capacity for the SN. Then, the causality constraint for battery-finite energy harvesting based wireless sensor networks is formulated as follows [4]:

(20a)P˜(η[t])≤∑i=0t−1H[i]−∑i=1t−1P˜(η[i]),∀t;(20b)∑i=0t−1H[i]−∑i=1t−1P˜(η[i])≤Bmax,∀t.

Based on Equation (20a,b), we can obtain that $\tilde{P}\left(\eta \left[t\right]\right)$ needs to satisfy:

(21) $$\begin{array}{c} \left\{\begin{array}{c} \tilde{P}\left(\eta \left[t\right]\right)\leq \sum_{i=0}^{t-1}H\left[i\right]-\sum_{i=1}^{t-1}\tilde{P}\left(\eta \left[i\right]\right),\;\forall t;\\ \tilde{P}\left(\eta \left[t\right]\right)\leq B_{\max},\;\forall t. \end{array}\right. \end{array}$$

Thus, when *t* approaches ∞, we can further simplify Equation (21) to the average harvested energy constraint and the battery capacity constraint as follows:

(22) $$\begin{array}{c} \left\{\begin{array}{c} E_{\gamma }\left(\tilde{P}\left(\eta \right)\right)\leq E_{H};\\ \tilde{P}\left(\eta \right)\leq B_{\max}. \end{array}\right. \end{array}$$

Now, we formulate the effective energy efficiency maximization problem for the battery-finite energy harvesting based wireless sensor networks as follows:

(23) $$P3:\arg\max_{\tilde{\mu }\left(\eta \right)}\left\{\frac{-\frac{1}{\theta }\log\int_{0}^{\infty }e^{-\theta T_{f}B\log_{2}\left(1+\tilde{\mu }\left(\eta \right)\gamma \right)}p_{\Gamma }\left(\gamma \right)d\gamma }{E_{H}}\right\},$$

subject to Equation (22).

It is hard to solve problem P3 since it is a non-convex optimization problem. Thus, we convert problem P3 into the equivalent problem P4 , which is a convex optimization problem, as follows:

(24) $$P4:\;\;\arg\min_{\tilde{\mu }\left(\eta \right)}\left\{\int_{0}^{\infty }{\left(1+\tilde{\mu }\left(\eta \right)\gamma \right)}^{-\beta }p_{\Gamma }\left(\gamma \right)d\gamma \right\},$$

subject to Equation (22).

Since the average harvested energy $E_{H}$ is variable in energy harvesting based wireless sensor networks, in order to solve the problem P4 , we need to analyze the cases that the optimal policy is determined by only the average harvested energy constraint ( $E_{\gamma }\left(\tilde{P}\left(\eta \right)\right)\leq E_{H}$ ), only the battery capacity constraint ( $\tilde{P}\left(\eta \right)\leq B_{\max}$ ), and both constraints specified in Equation (22).

### 4.2. The Optimal Power Control with QoS Provisioning in Battery-Finite Energy Harvesting Based Wireless Sensor Networks

If the battery capacity is large enough to store harvested energy without overflow, the optimal power control policy is not limited by the battery capacity. We denote by ${\hat{f}}_{\theta }\left(\lambda_{e}\right)$ the threshold to judge whether the battery capacity constraint is always satisfied or not (We will derive the closed-form expression for ${\hat{f}}_{\theta }\left(\lambda_{e}\right)$ in Section 4.3.). For fixed θ , if $B_{\max}\geq {\hat{f}}_{\theta }\left(\lambda_{e}\right)$ holds, the battery capacity constraint is always satisfied. In the case of $B_{\max}\geq {\hat{f}}_{\theta }\left(\lambda_{e}\right)$ , the optimal power control policy is only determined by average harvested energy constraint. Thus, the effective energy efficiency maximization problem P3 becomes problem P1 . Then, we give the following Proposition 1.

**Proposition** **1.** *If $B_{\max}\geq {\hat{f}}_{\theta }\left(\lambda_{e}\right)$ is satisfied, the optimal power control policy in battery-finite energy harvesting based wireless sensor networks is given as follows:*

(25) $$\begin{array}{c} {\tilde{\mu }}^{*}\left(\eta \right)=\left\{\begin{array}{cc} \frac{\lambda_{e}^{-\frac{1}{\beta +1}}}{\gamma_{\mathrm{in}}^{\frac{1}{\beta +1}}\gamma^{\frac{\beta }{\beta +1}}}-\frac{1}{\gamma }, & \gamma \geq \gamma_{\mathrm{in}};\\ 0, & \gamma <\gamma_{\mathrm{in}}. \end{array}\right. \end{array}$$

**Proof.**  The proof of Proposition 1 is very similar to the proof of Theorem 1. We omit the details here. ☐

If the transmitter always harvests energy more than the battery capacity, the energy overflowed will be wasted. In this case, the optimal power control policy is only determined by the battery capacity constraint. Thus, we have the following Proposition 2.

**Proposition** **2.** *If $B_{\max}\leq \lambda_{e}$ , the optimal power control policy in battery-finite energy harvesting based wireless sensor networks is given as follows:*

(26) $${\tilde{\mu }}^{*}\left(\eta \right)=\frac{B_{\max}}{\lambda_{e}}.$$

**Proof.**  If the optimal power control policy is only determined by the battery capacity constraint, the maximum available instantaneous power, denoted by $\tilde{P}\left(\eta \right)=B_{\max}$ , will be always optimal. Thus, in this case, the optimal power control policy is ${\tilde{\mu }}^{*}\left(\eta \right)=B_{\max}/\lambda_{e}$ . ☐

For the region $\lambda_{e}<B_{\max}<{\hat{f}}_{\theta }\left(\lambda_{e}\right)$ , the optimal power control policy is the solution of problem P4 . In this case, we solve problem P4 and have the following Theorem 2.

**Theorem** **2.** *If $\lambda_{e}<B_{\max}<{\hat{f}}_{\theta }\left(\lambda_{e}\right)$ is satisfied, the optimal power control policy in battery-finite energy harvesting based wireless sensor networks is given by*

(27) $$\begin{array}{c} {\tilde{\mu }}^{*}\left(\eta \right)=\left\{\begin{array}{cc} 0, & \gamma <\gamma_{\mathrm{fn}};\\ \frac{{\lambda_{e}}^{-\frac{1}{\beta +1}}}{\gamma_{\mathrm{fn}}^{\frac{1}{\beta +1}}\gamma^{\frac{\beta }{\beta +1}}}-\frac{1}{\gamma }, & \gamma \geq \gamma_{\mathrm{fn}}\;and\;f\left(\eta \right)\leq B_{\max};\\ \frac{B_{\max}}{\lambda_{e}}, & \gamma \geq \gamma_{\mathrm{fn}}\;and\;f\left(\eta \right)>B_{\max}, \end{array}\right. \end{array}$$

*where $f\left(\eta \right)≜{\lambda_{e}}^{\frac{\beta }{\beta +1}}/\left({\gamma_{\mathrm{fn}}}^{\frac{1}{\beta +1}}\gamma^{\frac{\beta }{\beta +1}}\right)-\lambda_{e}/\gamma$ is defined for simply expression and $\gamma_{\mathrm{fn}}$ is the cut-off SNR in battery-finite energy harvesting based wireless sensor networks. The parameter $\gamma_{\mathrm{fn}}$ can be numerically obtained by substituting Equation (27) into:*

(28) $$\int_{\gamma_{\mathrm{fn}}}^{\infty }{\tilde{\mu }}^{*}\left(\eta \right)p_{\Gamma }\left(\gamma \right)d\gamma =1.$$

**Proof.**  We formulate the Lagrangian function of problem P4 as follows:

(29) $$\begin{array}{c} L=\int_{0}^{\infty }{\left(1+\mu \left(\eta \right)\gamma \right)}^{-\beta }p_{\Gamma }\left(\gamma \right)d\gamma +\kappa_{1}\left(E_{\gamma }\left[P\left(\eta \right)\right]-\lambda_{e}\right)+\kappa_{2}\left(\lambda_{e}\mu \left(\eta \right)-B_{\max}\right), \end{array}$$

where $\kappa_{1}$ and $\kappa_{2}$ are the Lagrange multipliers corresponding to the constraints specified in Equation (22). Then, the corresponding KKT conditions can be expressed as follows:

(30) $$\left\{\begin{array}{c} -\beta \gamma {\left(1+\mu \left(\eta \right)\gamma \right)}^{-\beta -1}p_{\Gamma }\left(\gamma \right)+\kappa_{1}\lambda_{e}p_{\Gamma }\left(\gamma \right)+\kappa_{2}\lambda_{e}=0,\\ \kappa_{1}\left(\int_{0}^{\infty }\lambda_{e}\mu \left(\eta \right)p_{\Gamma }\left(\gamma \right)d\gamma -\lambda_{e}\right)=0,\\ \kappa_{2}\left(\lambda_{e}\mu \left(\eta \right)-B_{\max}\right)\geq 0,\\ \kappa_{1}\geq 0,\;\kappa_{2}\geq 0. \end{array}\right.$$

Solving Equation (30), we can obtain the optimal power control policy in Equation (27), where $\gamma_{\mathrm{fn}}≜\kappa_{1}/\beta$ and can be determined by the constraint Equation (28). ☐

**Figure 3:**
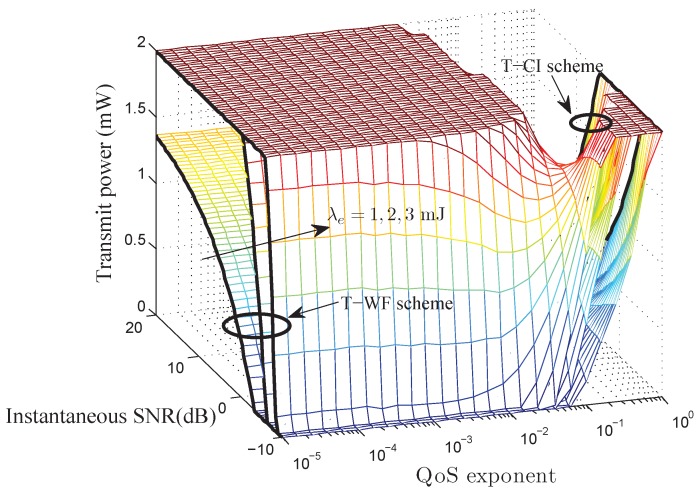
The QoS-driven power control policy for battery-finite energy harvesting based wireless sensor networks with m=2 , $\bar{\gamma }=5\;\mathrm{dB}$ , and $B_{\max}=2\;\mathrm{mJ}.$

Theorem 2 gives the QoS-driven power control policy for battery-finite energy harvesting based wireless sensor networks. According to the optimal power control policy given by Theorem 2, we plot the instantaneous power control policy corresponding to Equations (27) and (28) in Figure 3. As illustrated in Figure 3, for fixed energy arrival rate, the power control policy allocates more power to the better channel and less power to the worse channel when the QoS exponent is very small. When the QoS exponent is very large, the power control policy allocates more power to the worse channel and less power to the better channel. The allocated power increases as the energy arrival rate increases. Meanwhile, the cut-off SNR threshold $\gamma_{\mathrm{fn}}$ varies as the energy arrival rate varies. However, the maximum power is limited by the battery capacity. To further analyze the effect of QoS exponent on the optimal power control policy in battery-finite energy harvesting based wireless networks, we discuss two special cases of Theorem 2 in Remarks 3 and 4, which correspond to the optimal energy harvesting power control policies under the very loose QoS constraint and the very stringent QoS constraint, respectively.

**Remark** **3.** Under the very loose QoS constraint ( θ→0 ), the optimal power control policy ${\tilde{\mu }}^{*}\left(\eta \right)$ in Theorem 2 converges to

(31) $$\begin{array}{c} {\tilde{\mu }}^{*}\left(\eta \right)=\left\{\begin{array}{cc} 0, & \gamma <\gamma_{\mathrm{fn}};\\ \frac{1}{\lambda_{e}\gamma_{\mathrm{fn}}}-\frac{1}{\gamma }, & \gamma_{\mathrm{fn}}\leq \gamma <\hat{\gamma };\\ \frac{B_{\max}}{\lambda_{e}}, & \hat{\gamma }\leq \gamma , \end{array}\right. \end{array}$$

*where $\hat{\gamma }=\lambda_{e}\gamma_{\mathrm{fn}}/\left(1-B_{\max}\gamma_{\mathrm{fn}}\right)$ is the solution of $1/\left(\lambda_{e}\gamma_{\mathrm{fn}}\right)-1/\gamma =B_{\max}/\lambda_{e}$ . As θ varies to 0, the optimal power control policy in battery-finite energy harvesting based wireless sensor networks converges to the Truncated energy harvesting Water Filling (T-WF) scheme. In the T-WF scheme, both the energy arrival rate and the cut-off SNR threshold dominate the water level while the power is constrained by the battery capacity. The traditional directional water-filling scheme [4] is the special case ( θ=0 ) of the T-WF scheme.*

**Remark** **4.** Under the very stringent QoS constraint ( θ→∞ ), the optimal power control policy ${\tilde{\mu }}^{*}\left(\eta \right)$ in Theorem 2 converges to

(32) $$\begin{array}{c} {\tilde{\mu }}^{*}\left(\eta \right)=\left\{\begin{array}{cc} 0, & \gamma <\gamma_{\mathrm{fn}};\\ \frac{B_{\max}}{\lambda_{e}}, & \gamma_{\mathrm{fn}}\leq \gamma <\tilde{\gamma };\\ \frac{\sigma_{\mathrm{fn}}}{\gamma }, & \tilde{\gamma }\leq \gamma , \end{array}\right. \end{array}$$

*where $\sigma_{\mathrm{fn}}=\lim_{\theta \to \infty }\left[{\left(\lambda_{e}\gamma_{\mathrm{fn}}\right)}^{-\frac{1}{\beta +1}}-1\right]$ and $\tilde{\gamma }=\frac{\sigma_{\mathrm{fn}}\lambda_{e}}{B_{\max}}$ . Equation (32) represents that as θ approaches to ∞ the optimal power control policy in battery-finite energy harvesting system becomes the Truncated energy harvesting Channel Inversion (T-CI) scheme.*

As depicted in Figure 3, when the QoS exponent θ varies between 0 and ∞, the corresponding optimal power control policy for battery-finite energy harvesting based wireless sensor networks swings between the T-WF scheme and the T-CI scheme. Substituting Equations (27) and (28) into Equation (6), we can derive the maximum effective energy efficiency for battery-finite energy harvesting based wireless sensor networks, denoted by ${\tilde{E}}_{e}^{*}\left(\theta ,\lambda_{e}\right)$ , as follows:

(33) $$\begin{array}{cc} {\tilde{E}}_{e}^{*}\left(\theta ,\lambda_{e}\right)= & -\frac{1}{\theta \lambda_{e}}\log\{\frac{\gamma (m,\frac{m}{\bar{\gamma }}\gamma_{\mathrm{fn}})}{\Gamma (m)}\\ & +{\left\{{\left(\frac{\lambda_{e}m\gamma_{\mathrm{fn}}}{\bar{\gamma }}\right)}^{\frac{\beta }{\beta +1}}\frac{\Gamma (m-\frac{\beta }{\beta +1},\frac{m\gamma_{\mathrm{fn}}}{\bar{\gamma }})}{\Gamma (m)},\int_{\gamma_{\mathrm{fn}}}^{\infty }{\left[1+\frac{B_{\max}}{\lambda_{e}}\gamma \right]}^{-\beta }p_{\Gamma }\left(\gamma \right)d\gamma \right\}}^{+}\}, \end{array}$$

where ${\left\{a,b\right\}}^{+}≜\max\left\{a,b\right\}$ .

### 4.3. The Analysis for the Threshold of Energy Constraints ${\hat{f}}_{\theta }\left(\lambda_{e}\right)$

Based on the analyses of Section 4.2 for battery-finite energy harvesting based wireless sensor networks, if the optimal power control policy is only determined by the average harvested energy constraint, it needs to satisfy

(34) $$f\left(\eta \right)=\frac{{\lambda_{e}}^{\frac{\beta }{\beta +1}}}{{\gamma_{\mathrm{fn}}}^{\frac{1}{\beta +1}}\gamma^{\frac{\beta }{\beta +1}}}-\frac{\lambda_{e}}{\gamma }\leq B_{\max}.$$

To derive the maximum value of f(η) , which is ${\hat{f}}_{\theta }\left(\lambda_{e}\right)$ , we first check the convexity of function $f\left(\eta \right)={\lambda_{e}}^{\frac{\beta }{\beta +1}}/\left({\gamma_{\mathrm{fn}}}^{\frac{1}{\beta +1}}\gamma^{\frac{\beta }{\beta +1}}\right)-\lambda_{e}/\gamma$ by setting its secondary derivation with respect to γ to be 0 as follows:

(35) $$\frac{\partial^{2}f\left(\eta \right)}{\partial \gamma^{2}}=\frac{\beta \left(2\beta +1\right){\lambda_{e}}^{\frac{\beta }{\beta +1}}\gamma^{-\frac{3\beta +2}{\beta +1}}}{{\left(\beta +1\right)}^{2}{\gamma_{\mathrm{fn}}}^{\frac{1}{\beta +1}}}-2\lambda_{e}\gamma^{-3}=0.$$

Solving Equation (35), we can obtain $\gamma ={\left[\frac{2{\left(\beta +1\right)}^{2}}{\beta (2\beta +1)}\right]}^{\beta +1}\lambda_{e}\gamma_{\mathrm{fn}}$ . For the region $\gamma <{\left[\frac{2{\left(\beta +1\right)}^{2}}{\beta (2\beta +1)}\right]}^{\beta +1}\lambda_{e}\gamma_{\mathrm{fn}}$ , $\partial^{2}f\left(\eta \right)/\partial \gamma^{2}$ is less than zero corresponding to the low SNR region. When $\gamma \geq {\left[\frac{2{\left(\beta +1\right)}^{2}}{\beta (2\beta +1)}\right]}^{\beta +1}\lambda_{e}\gamma_{\mathrm{fn}}$ , $\partial^{2}f\left(\eta \right)/\partial \gamma^{2}$ is larger than or equal to zero corresponding to the high SNR region. Thus, f(η) is concave in the low SNR region and convex in the high SNR region. We set the first derivation to zero as follows:

(36) $$\frac{\partial f(\eta )}{\partial \gamma }=-\frac{\beta {\lambda_{e}}^{\frac{\beta }{\beta +1}}\gamma^{-\frac{2\beta +1}{\beta +1}}}{\left(\beta +1\right)\gamma_{\mathrm{fn}}^{\frac{1}{\beta +1}}}+\lambda_{e}\gamma^{-2}=0,$$

solving which, we can obtain the stationary point as follows:

(37) $$\gamma ={\left(\frac{\beta +1}{\beta }\right)}^{\beta +1}\lambda_{e}\gamma_{\mathrm{fn}}.$$

Because of ${\left[\frac{\beta +1}{\beta }\right]}^{\beta +1}\lambda_{e}\gamma_{\mathrm{fn}}<{\left[\frac{2{\left(\beta +1\right)}^{2}}{\beta (2\beta +1)}\right]}^{\beta +1}\lambda_{e}\gamma_{\mathrm{fn}}$ , the stationary point falls into the low SNR region. Therefore, the maximum of f(η) in the low SNR region corresponds to the stationary point $\gamma ={\left(\frac{\beta +1}{\beta }\right)}^{\beta +1}\lambda_{e}\gamma_{\mathrm{fn}}$ . Then, substituting Equation (37) into the function of f(η) specified in Equation (34), we can obtain that, in the low SNR region, f(η) needs to satisfy

(38) $$f\left(\eta \right)\leq \frac{\beta^{\beta }}{\gamma_{\mathrm{fn}}{\left(\beta +1\right)}^{\beta +1}}.$$

In the high SNR region, since f(η) is convex, the maximum of f(η) can be obtained between the following two boundary points:

(39) $$\left\{\begin{array}{c} \gamma ={\left[\frac{2{\left(\beta +1\right)}^{2}}{\beta (2\beta +1)}\right]}^{\beta +1}\lambda_{e}\gamma_{\mathrm{fn}};\\ \gamma =\infty . \end{array}\right.$$

Substituting the two functions in Equation (39) into Equation (34), respectively, we can derive that in the high SNR region f(η) needs to satisfy:

(40) $$\begin{array}{c} f\left(\eta \right)\leq \max\left\{\frac{\beta^{\beta }{\left(2\beta +1\right)}^{\beta (3\beta +2)}}{\gamma_{\mathrm{fn}}{\left[2{\left(\beta +1\right)}^{2}\right]}^{\beta +1}},0\right\}=\frac{\beta^{\beta }{\left(2\beta +1\right)}^{\beta (3\beta +2)}}{\gamma_{\mathrm{fn}}{\left[2{\left(\beta +1\right)}^{2}\right]}^{\beta +1}}. \end{array}$$

Then, based on Equations (38) and (40), the upper bound of f(η) is given as follows:

(41) $$\begin{array}{c} f\left(\eta \right)\leq \max\left\{\frac{\beta^{\beta }}{\gamma_{\mathrm{fn}}{\left(\beta +1\right)}^{\beta +1}},\frac{\beta^{\beta }{\left(2\beta +1\right)}^{\beta (3\beta +2)}}{\gamma_{\mathrm{fn}}{\left[2{\left(\beta +1\right)}^{2}\right]}^{\beta +1}}\right\}=\frac{\beta^{\beta }}{\gamma_{\mathrm{fn}}{\left(\beta +1\right)}^{\beta +1}}, \end{array}$$

where the equality holds for the reason that function f(η) is continuous and, in the low SNR region, the value at the stationary point is larger than the value at the inflection point. Therefore, we can obtain the closed-form of ${\hat{f}}_{\theta }\left(\lambda_{e}\right)$ as :

(42) $${\hat{f}}_{\theta }\left(\lambda_{e}\right)=\frac{\beta^{\beta }}{\gamma_{\mathrm{fn}}{\left(\beta +1\right)}^{\beta +1}}.$$

As a result, if $B_{\max}\geq \beta^{\beta }/\gamma_{\mathrm{fn}}{\left(\beta +1\right)}^{\beta +1}$ holds, the battery capacity constraint is always satisfied.

## 5. Outage Probability Analyses

For energy harvesting based wireless networks, there exits the energy outage probability and the data-transmission outage probability [35,36]. The energy outage probability is the probability that harvested energy is not sufficient enough to keep the power consumption, i.e., $\sum_{i=1}^{t}P\left(\eta \left[i\right]\right)\geq \sum_{i=0}^{t-1}H\left[i\right]$ . The data-transmission outage probability is the probability that instantaneous service rate cannot support the required target data rate. Let $P_{\mathrm{out}}^{e}$ and $P_{\mathrm{out}}^{d}$ denote by the energy outage probability and data-transmission outage probability, respectively. In the following, we analyze the energy outage probability and data-transmission outage probability, respectively, to theoretically evaluate the performance for energy harvesting based wireless sensor networks.

### 5.1. Energy Outage Probability

For energy harvesting based wireless sensor networks, we have the following Lemma 1 regarding the energy outage probability.

**Lemma** **1.** *When t approaches to ∞, $P_{\mathrm{out}}^{e}$ converges to 0.*

**Proof.**  Using our developed optimal power control policies, the energy outage probability for energy harvesting based wireless sensor networks can be derived as follows:

(43) $$\begin{array}{c} P_{\mathrm{out}}^{e}=\mathrm{Pr}\left\{\sum_{i=1}^{t}P^{*}\left(\eta \left[i\right]\right)-\sum_{i=0}^{t-1}H\left[i\right]\geq 0\right\}=\mathrm{Pr}\left\{\sum_{i=1}^{t}P^{*}\left(\eta \left[i\right]\right)-\sum_{i=1}^{t-1}H\left[i\right]\geq H\left[0\right]\right\},\;\forall t, \end{array}$$

where $P^{*}\left(\eta \left[i\right]\right)=\mu^{*}\left(\eta \left[i\right]\right)\lambda_{e}$ denotes the optimal power allocation in the $i^{\mathrm{th}}$ frame. According to Equations (14) and (28), $P^{*}\left(\eta \left[i\right]\right)$ needs to satisfy

(44) $$E_{\gamma }\left[P^{*}\left(\eta \right)\right]=E_{H}.$$

Thus, when *t* approaches to ∞, the expectation of $\sum_{i=1}^{t}P^{*}\left(\eta \left[i\right]\right)$ is equivalent to the expectation of $\sum_{i=1}^{t}H\left[i\right]$ and can be written as follows:

(45) $$E\left[\sum_{i=1}^{t}P^{*}\left(\eta \left[i\right]\right)\right]=E\left[\sum_{i=1}^{t}H\left[i\right]\right].$$

Based on Equations (43) and (45), and the law of Chebyshev large numbers [37], we can obtain

(46) $$\lim_{t\to \infty }\mathrm{Pr}\left\{\frac{1}{t}\sum_{i=1}^{t}P^{*}\left(\eta \left[i\right]\right)-\frac{1}{t}\sum_{i=1}^{t}H\left[i\right]\geq \varepsilon \right\}=0.$$

Let $\varepsilon ≜\lim_{t\to \infty }\frac{1}{t}\left(H\left[0\right]-H\left[t\right]\right)$ . We can convert Equation (46) as follows:

(47) $$\lim_{t\to \infty }P_{\mathrm{out}}^{e}\;\;=\;\;\lim_{t\to \infty }\mathrm{Pr}\left\{\frac{1}{t}\left[\sum_{i=1}^{t}P^{*}\left(\eta \left[i\right]\right)-\sum_{i=0}^{t-1}H\left[i\right]\right]\geq 0\right\}=0,$$

which shows the energy outage probability converges to zero as *t* approaches to ∞. ☐

Now, we have derived that $P_{\mathrm{out}}^{e}$ converges to zero when *t* approaches to infinity. Next, when *t* is not infinite, we can derive the upper-bound for the energy outage probability according to the Chebyshev inequality [37] as follows:

(48) $$\begin{array}{c} P_{\mathrm{out}}^{e}=\mathrm{Pr}\left\{\sum_{i=1}^{t}P^{*}\left(\eta \left[i\right]\right)-\sum_{i=1}^{t}H\left[i\right]\geq H\left[0\right]-H\left[t\right]\right\}\leq \frac{D\left\{\sum_{i=1}^{t}P^{*}\left(\eta \left[i\right]\right)\right\}}{{\left(H\left[0\right]-H\left[t\right]\right)}^{2}},\;\forall t, \end{array}$$

where D[a] represents the variance of *a*.

Observing Equation (48), we find that $P_{\mathrm{out}}^{e}$ decreases as H[0] increases. Moreover, according to Lemma 1, $P_{\mathrm{out}}^{e}$ converges to 0 when *t* approaches to ∞. Practically, it always needs to take a relatively long time to cumulate energy from the energy sources before starting communications. Therefore, the energy outage probability can be regarded as zero by charging the battery for a while in reality.

### 5.2. Data-Transmission Outage Probability

Using our developed optimal power control policies, the data-transmission outage probability for energy harvesting based wireless sensor networks can be formulated as follows [38]:

(49) $$\begin{array}{c} P_{\mathrm{out}}^{d}=\mathrm{Pr}\left\{T_{f}B\log_{2}\left(1+\mu^{*}\gamma \right)\leq R_{\mathrm{th}}\right\}=\mathrm{Pr}\left\{\gamma \leq \frac{2^{\frac{R_{\mathrm{th}}}{T_{f}B}}-1}{\mu^{*}}\right\}, \end{array}$$

where $R_{\mathrm{th}}$ is the required target service rate. Based on the work of [39,40], the data-transmission outage probability in Equation (49) can be converted as follows:

(50) $$P_{\mathrm{out}}^{d}=1-\exp\left[-{\left(\frac{2^{\frac{R_{\mathrm{th}}}{T_{f}B}}-1}{\mu^{*}}\right)}^{\frac{\alpha }{2}}\right],$$

where α is the parameter controlling the severity or the diversity of the channel fading. Then, we analyze the data-transmission outage probabilities in battery-infinite and battery-finite energy harvesting based wireless networks, respectively.

#### 5.2.1. Battery-Infinite Energy Harvesting Based Wireless Sensor Networks

The optimal power control policy for battery-infinite energy harvesting based wireless networks has been shown in Theorem 1. Plugging Equation (13) into Equation (50), we can obtain the data-transmission outage probability, denoted by $P_{\mathrm{out}}^{d_{i}}$ , for battery-infinite energy harvesting based wireless sensor networks as follows:

(51) $$P_{\mathrm{out}}^{d_{i}}=1-\exp\left[-{\left(\frac{\left(2^{\frac{R_{\mathrm{th}}}{T_{f}B}}-1\right)\gamma_{\mathrm{in}}^{\frac{1}{\beta +1}}\gamma }{{\left(\lambda_{e}^{-1}\gamma \right)}^{\frac{1}{\beta +1}}-\gamma_{\mathrm{in}}^{\frac{1}{\beta +1}}}\right)}^{\frac{\alpha }{2}}\right],\gamma \geq \gamma_{\mathrm{in}}.$$

To further evaluate the data-transmission outage probability, we obtain Lemma 2 regarding $P_{\mathrm{out}}^{d_{i}}$ under two specified cases, i.e., when QoS constraint is very loose and QoS constraint is very stringent.

**Lemma** **2.** *When the QoS constraint is very loose ( θ→0 ), the data-transmission outage probability for battery-infinite energy harvesting based wireless sensor networks converges to*

(52) $$P_{\mathrm{out}}^{d_{i}}=\left\{\begin{array}{cc} 1, & \gamma \to 0;\\ 1-\exp\left[{\left(\left(2^{\frac{R_{\mathrm{th}}}{T_{f}B}}-1\right)\lambda_{e}\gamma_{\mathrm{in}}\right)}^{\frac{\alpha }{2}}\right], & \gamma \to \infty . \end{array}\right.$$

*When the QoS constraint is very stringent ( θ→∞ ), the data-transmission outage probability for battery-infinite energy harvesting based wireless sensor networks converges to*

(53) $$P_{\mathrm{out}}^{d_{i}}=\left\{\begin{array}{cc} 0, & \gamma \to 0;\\ 1, & \gamma \to \infty . \end{array}\right.$$

**Proof.**  Based on Equation (51), we analyze $P_{\mathrm{out}}^{d_{i}}$ in the following two cases corresponding to the data-transmission outage probabilities, under the very loose QoS constraint and the very stringent QoS constraint, respectively.

Case I: Under the very loose QoS constraint ( θ→ 0), the data-transmission outage probability for battery-infinite energy harvesting based wireless sensor networks converges to

(54) $$P_{\mathrm{out}}^{d_{i}}=1-\exp\left[-{\left(\frac{2^{\frac{R_{\mathrm{th}}}{T_{f}B}}-1}{{\left(\lambda_{e}\gamma_{\mathrm{in}}\right)}^{-1}-\gamma^{-1}}\right)}^{\frac{\alpha }{2}}\right],\gamma \geq \gamma_{\mathrm{in}}.$$

In this case, $P_{\mathrm{out}}^{d_{i}}$ converges to 1 as γ approaches to zero. $P_{\mathrm{out}}^{d_{i}}$ becomes $1-\exp\left[{\left(\left(2^{\frac{R_{\mathrm{th}}}{T_{f}B}}-1\right)\lambda_{e}\gamma_{\mathrm{in}}\right)}^{\frac{\alpha }{2}}\right]$ as γ approaches to ∞.

Case II: Under the very stringent QoS constraint ( θ→∞ ), the data-transmission outage probability for battery-infinite energy harvesting based wireless sensor networks becomes

(55) $$P_{\mathrm{out}}^{d_{i}}=1-\exp\left[-{\left(\frac{2^{\frac{R_{\mathrm{th}}}{T_{f}B}}-1}{\sigma_{\mathrm{in}}\gamma^{-1}}\right)}^{\frac{\alpha }{2}}\right],\gamma \geq \gamma_{\mathrm{in}}.$$

Observing Equation (55), we find that $P_{\mathrm{out}}^{d_{i}}$ converges to zero as γ approaches to zero. Meanwhile, $P_{\mathrm{out}}^{d_{i}}$ becomes 1 as γ approaches to ∞.

Therefore, comprehensively considering both Cases I and II, we have Lemma 2. ☐

Based on the proof of Lemma 2, we can also obtain that under the very loose QoS constraint, $P_{\mathrm{out}}^{d_{i}}$ decreases as γ increases. Meanwhile, under the very stringent QoS constraint, $P_{\mathrm{out}}^{d_{i}}$ increases as γ increases.

#### 5.2.2. Battery-Finite Energy Harvesting Based Wireless Sensor Networks

Substituting Equation (27) into Equation (50), we can obtain the data-transmission outage probability, denoted by $P_{\mathrm{out}}^{d_{f}}$ , for battery-finite energy harvesting based wireless sensor networks as follows:

(56) $$P_{\mathrm{out}}^{d_{f}}=\left\{\begin{array}{c} 1-\exp\left[-{\left(\frac{\left(2^{\frac{R_{\mathrm{th}}}{T_{f}B}}-1\right)\gamma_{\mathrm{fn}}^{\frac{1}{\beta +1}}\gamma }{{\left(\lambda_{e}^{-1}\gamma \right)}^{\frac{1}{\beta +1}}-\gamma_{\mathrm{fn}}^{\frac{1}{\beta +1}}}\right)}^{\frac{\alpha }{2}}\right],\gamma \geq \gamma_{\mathrm{fn}}\;\mathrm{and}\;f\left(\eta \right)<B_{\max};\\ 1-\exp\left[-{\left(\frac{\left(2^{\frac{R_{\mathrm{th}}}{T_{f}B}}-1\right)\lambda_{e}}{B_{\max}}\right)}^{\frac{\alpha }{2}}\right],\gamma \geq \gamma_{\mathrm{fn}}\;\mathrm{and}\;f\left(\eta \right)>B_{\max}. \end{array}\right.$$

Then, we obtain the upper and lower bounds of $P_{\mathrm{out}}^{d_{f}}$ under the very loose QoS constraint and the very stringent QoS constraint, respectively, in Lemma 3.

**Lemma** **3.** *When the QoS constraint is very loose ( θ→0 ), the data-transmission outage probability for battery-finite energy harvesting based wireless sensor networks converges to*

(57) $$P_{\mathrm{out}}^{d_{f}}=\left\{\begin{array}{cc} 1, & \gamma \to 0;\\ 1-\exp\left[-{\left(\frac{\left(2^{\frac{R_{\mathrm{th}}}{T_{f}B}}-1\right)\lambda_{e}}{B_{\max}}\right)}^{\frac{\alpha }{2}}\right], & \gamma \to \infty . \end{array}\right.$$

*When the QoS constraint is very stringent ( θ→∞ ), the data-transmission outage probability for battery-finite energy harvesting based wireless sensor networks converges to*

(58) $$P_{\mathrm{out}}^{d_{f}}=\left\{\begin{array}{cc} 1-\exp\left[-{\left(\frac{\left(2^{\frac{R_{\mathrm{th}}}{T_{f}B}}-1\right)\lambda_{e}}{B_{\max}}\right)}^{\frac{\alpha }{2}}\right], & \gamma \to 0;\\ 1, & \gamma \to \infty . \end{array}\right.$$

**Proof.**  The expression of $P_{\mathrm{out}}^{d_{f}}$ has been specified in Equation (56). Then, we analyze the data-transmission outage probability for battery-finite energy harvesting based wireless sensor networks in two specific cases corresponding to the data-transmission outage probabilities under the very loose QoS constraint and the very stringent QoS constraint, respectively.

Case 1: Under the very loose QoS constraint ( θ→0 ), the data-transmission outage probability for battery-finite energy harvesting based wireless sensor networks converges to

(59) $$P_{\mathrm{out}}^{d_{f}}=\left\{\begin{array}{cc} 1-\exp\left[-{\left(\frac{2^{\frac{R_{\mathrm{th}}}{T_{f}B}}-1}{{\left(\lambda_{e}\gamma_{\mathrm{fn}}\right)}^{-1}-\gamma^{-1}}\right)}^{\frac{\alpha }{2}}\right], & \gamma_{\mathrm{fn}}\leq \gamma <\hat{\gamma };\\ 1-\exp\left[-{\left(\frac{\left(2^{\frac{R_{\mathrm{th}}}{T_{f}B}}-1\right)\lambda_{e}}{B_{\max}}\right)}^{\frac{\alpha }{2}}\right], & \hat{\gamma }\leq \gamma . \end{array}\right.$$

Observing Equation (59), we find that $P_{\mathrm{out}}^{d_{f}}$ turns to 1 when γ approaches to zero. $P_{\mathrm{out}}^{d_{f}}$ converges to $1-\exp[-{\left(\left(2^{\frac{R_{\mathrm{th}}}{T_{f}B}}-1\right)\lambda_{e}/B_{\max}\right)}^{\frac{\alpha }{2}}]$ when γ approaches to ∞.

Case 2: Under the very stringent QoS constraint ( θ→∞ ), the data-transmission outage probability for battery-finite energy harvesting based wireless sensor networks converges to:

(60) $$P_{\mathrm{out}}^{d_{f}}=\left\{\begin{array}{cc} 1-\exp\left[-{\left(\frac{\left(2^{\frac{R_{\mathrm{th}}}{T_{f}B}}-1\right)\lambda_{e}}{B_{\max}}\right)}^{\frac{\alpha }{2}}\right], & \gamma_{\mathrm{fn}}\leq \gamma <\hat{\gamma };\\ 1-\exp\left[-{\left(\frac{2^{\frac{R_{\mathrm{th}}}{T_{f}B}}-1}{\sigma_{\mathrm{fn}}\gamma^{-1}}\right)}^{\frac{\alpha }{2}}\right], & \hat{\gamma }\leq \gamma . \end{array}\right.$$

Based on Equation (60), we can obtain that $P_{\mathrm{out}}^{d_{f}}$ converges to $1-\exp[-{\left(\left(2^{\frac{R_{\mathrm{th}}}{T_{f}B}}-1\right)\lambda_{e}/B_{\max}\right)}^{\frac{\alpha }{2}}]$ when γ approaches to zero. Meanwhile, $P_{\mathrm{out}}^{d_{f}}$ turns to 1 when γ approaches to ∞.

Thus, based on the analyses for Cases 1 and 2, we have Lemma 3. ☐

Equations (59) and (60) show that under the very loose QoS constraint, $P_{\mathrm{out}}^{d_{f}}$ decreases as γ increases. Under the very stringent QoS constraint, $P_{\mathrm{out}}^{d_{f}}$ increases as γ increases.

Since the energy outage probability can be treated as zero, the outage probability for energy harvesting based wireless sensor networks can be entirely determined by the data-transmission outage probability, which is calculated based on Equations (51) and (56). Both Equations (51) and (56) show that the outage probabilities are functions of instantaneous SNR γ , QoS constraint θ , and energy arrival rate $\lambda_{e}$ . Based on Equations (51) and (56), we can derive the outage probability corresponding to the specified instantaneous SNR, QoS constraint, and energy arrival rate.

## 6. Performance Evaluation

In this section, we conduct numerical analyses to evaluate the performance of our proposed QoS-driven power control policies for energy harvesting based wireless sensor networks. Throughout the simulation, we use normalized effective energy efficiency and normalized effective capacity (EC), which are defined as the EEE and EC per Hz per second, respectively, to evaluate the performance of the energy harvesting based wireless networks. We also set the bandwidth, the time frame length, the maximum battery capacity and the parameters of Nakagami-*m* channel model to be B=1MHz , $T_{f}=0.2\;\mathrm{ms}$ , $B_{\max}=2\;\mathrm{mJ}$ , $\bar{\gamma }=5\;\mathrm{dB}$ , and m=2 .

**Figure 4:**
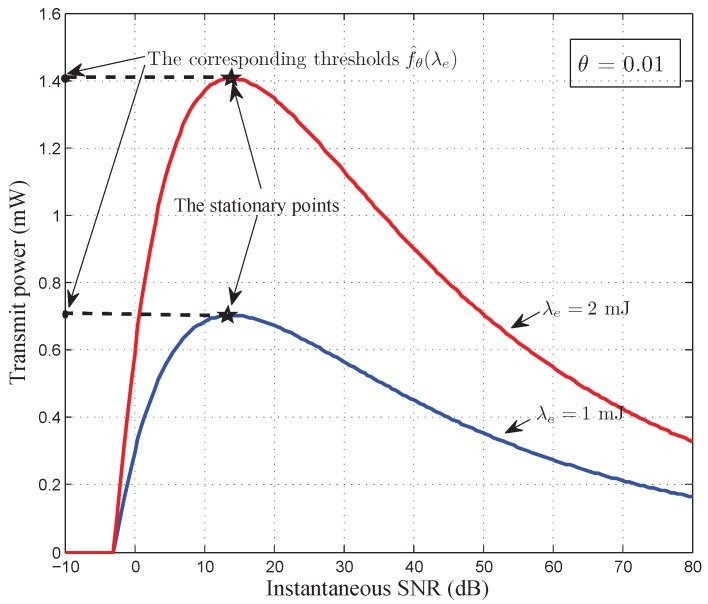
The transmit power curves versus instantaneous SNR to show the threshold ${\hat{f}}_{\theta }\left(\lambda_{e}\right)$ under θ = 0.01.

**Figure 5:**
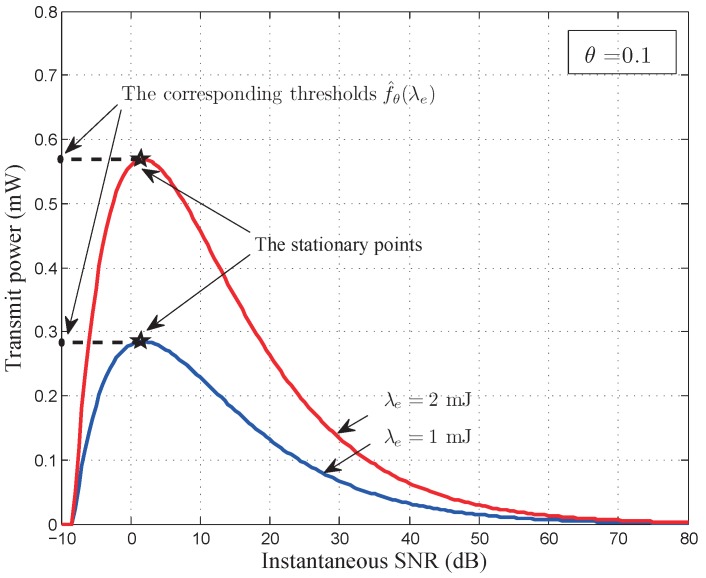
The transmit power curves versus instantaneous SNR to show the threshold ${\hat{f}}_{\theta }\left(\lambda_{e}\right)$ under θ = 0.1.

In order to numerically analyze the threshold ${\hat{f}}_{\theta }\left(\lambda_{e}\right)$ for energy constraints, we plot the transmit power curves versus the instantaneous SNR in Figure 4 and Figure 5, where the QoS constraint θ is set to be 0.01 and 0.1, respectively. Observing Figure 4 and Figure 5, we find that the transmit power curves are concave when γ is very small and convex when γ is very large. This validates our analyses for threshold ${\hat{f}}_{\theta }\left(\lambda_{e}\right)$ of energy constraints in Section 4.3. The maximum value of transmit power, which corresponds to the thresholds ${\hat{f}}_{\theta }\left(\lambda_{e}\right)$ , can be obtained at the stationary points in Figure 4 and Figure 5, i.e., when θ=0.01 and $\lambda_{e}=2$ , ${\hat{f}}_{0.01}\left(2\right)=1.406$ , which represents that if $B_{\max}\geq 1.406$ , the optimal power control policy is dominated only by the average harvested energy constraint under this circumstance. Figure 4 and Figure 5 also illustrate that, for different energy arrival rates and under different QoS constraints, we can obtain different energy constraints’ thresholds ${\hat{f}}_{\theta }\left(\lambda_{e}\right)$ . This verifies that ${\hat{f}}_{\theta }\left(\lambda_{e}\right)$ depends on the energy arrival rate $\lambda_{e}$ and QoS constraint θ .

**Figure 6:**
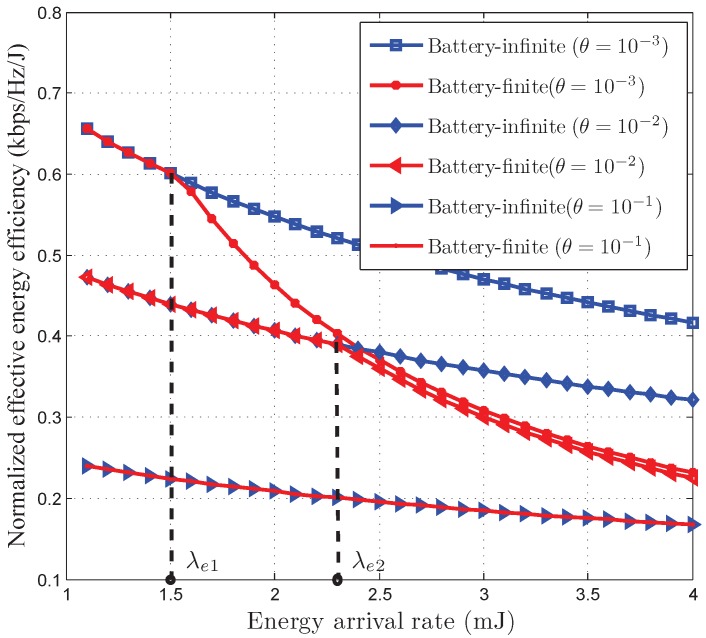
The normalized effective energy efficiency of our developed QoS-driven power control policies versus energy arrival rate for battery-infinite/finite energy harvesting based wireless sensor networks.

**Figure 7:**
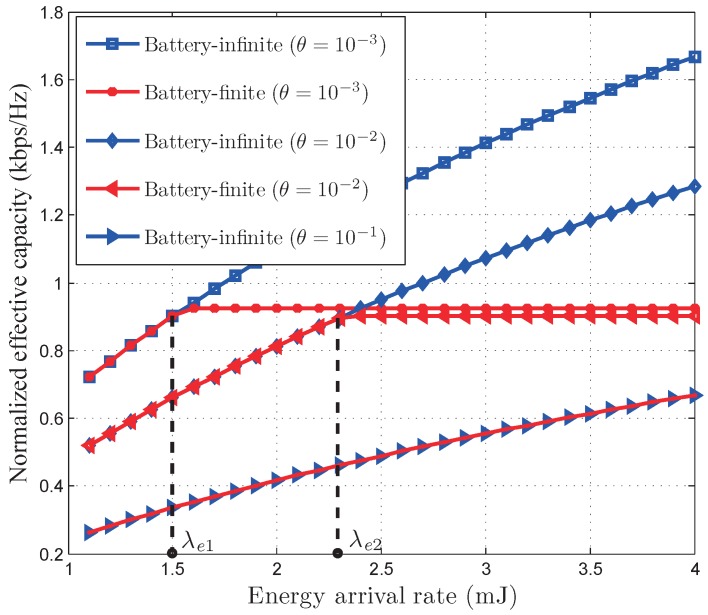
The normalized effective capacity of our developed QoS-driven power control policies versus energy arrival rate for battery-infinite/finite energy harvesting based wireless sensor networks.

Figure 6 and Figure 7 depict the normalized EEE and the normalized EC of our developed optimal power control policy versus energy arrival rate $\lambda_{e}$ . As illustrated in Figure 6 and Figure 7, EEE decreases as energy arrival rata increases while EC increases as energy arrival rate increases. This indicates that there is a trade-off between the EEE and EC. Also illustrated in Figure 6 and Figure 7, for $\lambda_{e}\leq \lambda_{e1}$ (under the QoS constraint $\theta =10^{-3}$ ) and $\lambda_{e}\leq \lambda_{e2}$ (under the QoS constraint $\theta =10^{-2}$ ), respectively, both the optimal power control policies in battery-infinite and battery-finite energy harvesting based wireless sensor networks have the same EEE and EC. This is because the instantaneous power control policy given by Proposition 1 is only limited by average harvested energy in the low energy arrival rate region. Therefore, when $\lambda_{e}\leq \lambda_{e1}$ (under the QoS constraint $\theta =10^{-3}$ ) and $\lambda_{e}\leq \lambda_{e2}$ (under the QoS constraint $\theta =10^{-2}$ ), the EEE and EC are not limited by the battery capacity. However, the battery capacity limits the EEE and EC in the high energy arrival rate region. For this reason, the optimal power control policy for battery-infinite energy harvesting based wireless sensor networks achieves much larger EEE and EC than that for battery-finite energy harvesting based wireless sensor networks when $\lambda_{e}>\lambda_{e1}$ (under the QoS constraint $\theta =10^{-3}$ ) and $\lambda_{e}>\lambda_{e2}$ (under the QoS constraint $\theta =10^{-2}$ ). We can also observe from Figure 6 and Figure 7 that, under the QoS constraint $\theta =10^{-1}$ , both the the battery-infinite and battery-finite energy harvesting based wireless sensor networks have the same EEE and EC when $\lambda_{e}$ is less than 4. This indicates that, when the QoS constraint is very stringent, the optimal power control policy for battery-finite energy harvesting based wireless sensor networks is not limited by battery capacity until the networks have a relatively large energy arrival rate.

**Figure 8:**
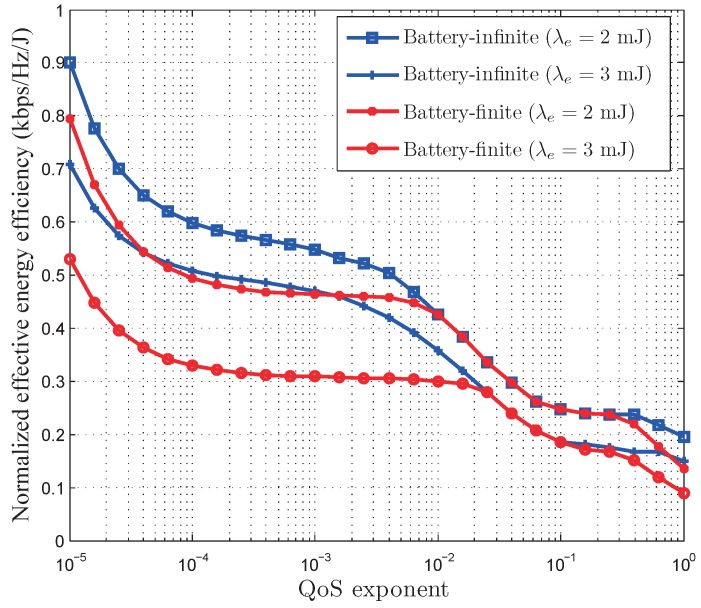
The normalized effective energy efficiency of our developed QoS-driven power control policies versus QoS exponent in battery-infinite and battery-finite energy harvesting based wireless sensor networks.

Figure 8 depicts the normalized EEE of the optimal power control policy versus the QoS exponent, where the energy arrival rate $\lambda_{e}$ is fixed to 2 and 3, respectively. As shown in Figure 8, the normalized EEE decreases as the QoS exponent θ increases. This indicates that the looser the traffic QoS constraint is, the larger EEE we can achieve. In addition, the optimal power control policy in battery-infinite energy harvesting based wireless sensor networks can achieve larger EEE than that in battery-finite energy harvesting based wireless sensor networks when the QoS constraint is very loose or very stringent. This is due to the reason that the QoS-driven power control policy in battery-finite energy harvesting based wireless sensor networks is limited by the battery capacity in the high SNR region when the QoS requirement is very loose and in the low SNR region when the QoS constraint is very stringent. When the QoS constraint is not very loose or not very stringent, both the QoS-driven power control policies for the battery-infinite and battery-finite energy harvesting based wireless sensor networks have the same EEE. This is because the maximum instantaneous transmit power is always less than the battery capacity when the QoS constraint is not very loose or not very stringent.

**Figure 9:**
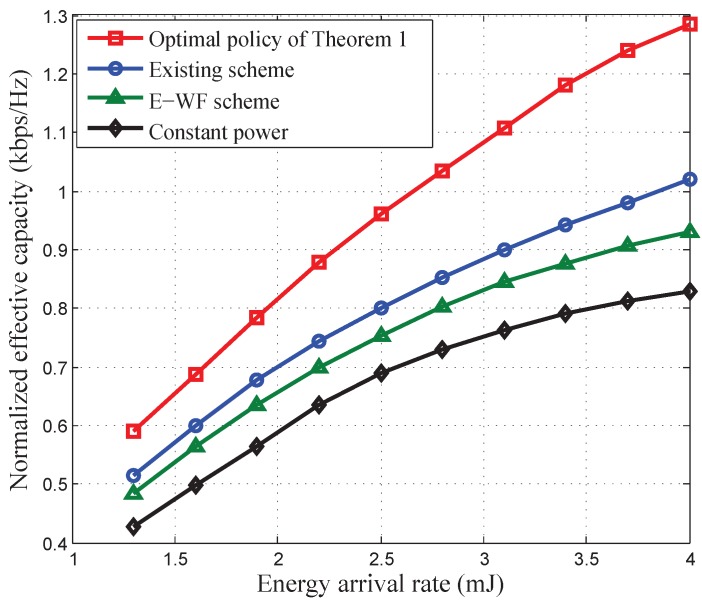
The comparison between our developed QoS-driven optimal power control policy, the existing related scheme, E-WF scheme, and constant power allocation scheme.

**Figure 10:**
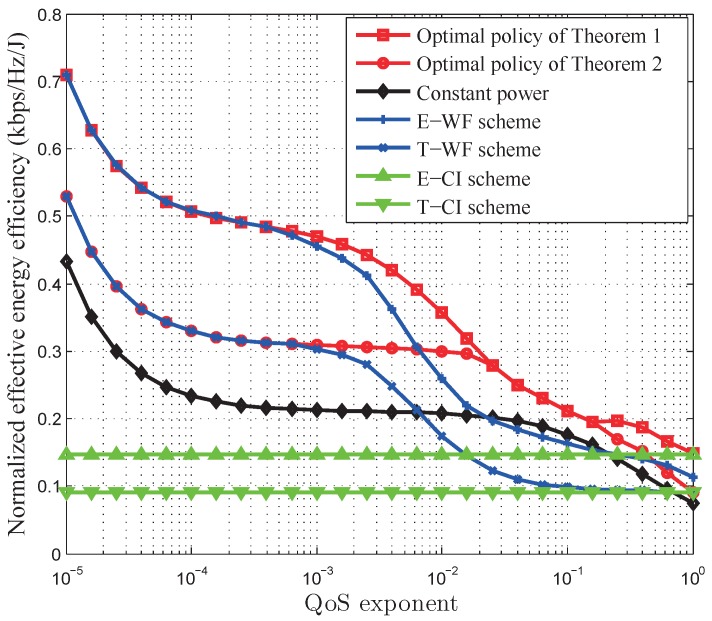
The comparison between our developed QoS-driven optimal power control policies, the constant power allocation scheme, the E-WF scheme, T-WF scheme, E-CI scheme, and T-CI scheme.

Figure 9 compares the performance of our developed optimal power control policy with other existing schemes, i.e., the related research works [25], E-WF scheme, and constant power allocation scheme. We find that both the power control policies with QoS provisioning specified in this paper and [25] can achieve better performance than the power control policies without QoS provisioning, i.e., the E-WF scheme and the constant power allocation. In addition, Figure 9 also shows that our developed optimal power control policy in Theorem 1 can achieve larger EC than the power control policy in [25]. This is because in [25] the data rate QoS requirement is considered, which is deterministic QoS, while our developed optimal power control policy provides the statistical QoS guarantees, which is adaptive to diverse delay-bounded QoS constraints, thus achieving the maximum EC. To further verify the analyses in this paper, we plot normalized EEE of the optimal power control polices developed in Theorems 1 and 2, constant power allocation, E-WF scheme, T-WF scheme, E-CI scheme, and T-CI scheme in Figure 10. We can observe that our developed QoS-driven power control policies, which are the solution of Theorems 1 and 2, can achieve larger EEE than other schemes for energy harvesting based wireless sensor networks. When the QoS constraint is very loose, our developed QoS-driven power control policy for battery-infinite energy harvesting based wireless sensor networks converges to the E-WF scheme and our developed QoS-driven power control policy for battery-finite energy harvesting based wireless sensor networks converges to the T-WF scheme. When the QoS requirement is very stringent, our QoS-driven optimal power control policy for battery-infinite energy harvesting based wireless sensor networks converges to the E-CI scheme and the QoS-driven power control policy for battery-finite energy harvesting based wireless sensor networks converges to the T-CI scheme.

**Figure 11:**
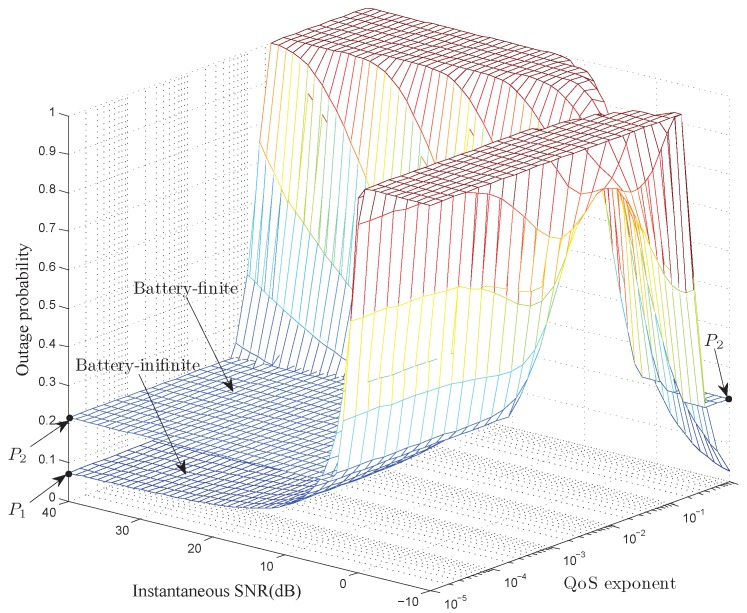
The outage probability of our developed optimal power control policy with α=4 , $\lambda_{e}=3\;\mathrm{mJ}$ , and $B_{\max}=1.5\;\mathrm{mJ}$ .

**Figure 12:**
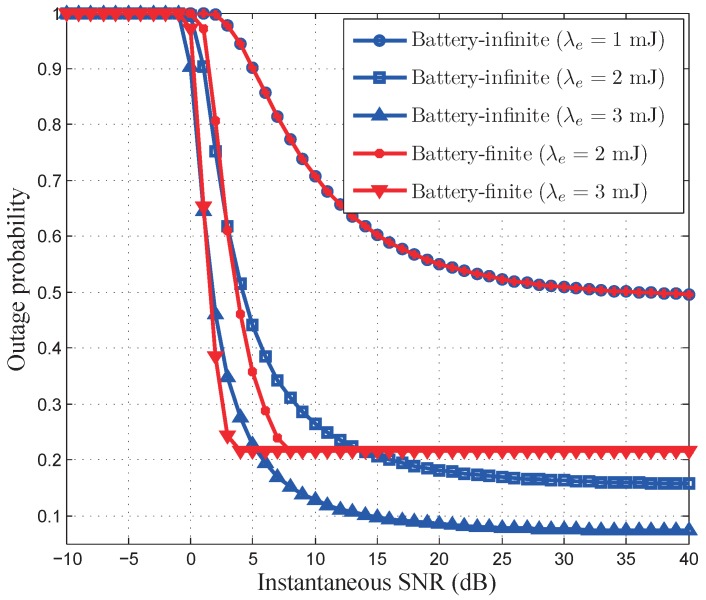
The outage probability versus instantaneous SNR under different energy arrival rates with θ = $10^{-4}.$

Figure 11 and Figure 12 illustrate the outage probabilities of our developed optimal power control policies. As depicted in Figure 11, when the QoS exponent θ is very small, the outage probability for battery-infinite energy harvesting based wireless sensor networks converges to 1 in the low SNR region and $P_{1}$ in the high SNR region, while the outage probability for battery-finite energy harvesting based wireless sensor networks converges to 1 in the low SNR region and $P_{2}$ in the high SNR region. In addition, when the QoS exponent θ is very large, the outage probability for battery-infinite energy harvesting based wireless sensor networks converges to zero in the low SNR region and 1 in the high SNR region, while the outage probability for battery-finite energy harvesting based wireless sensor networks converges to $P_{2}$ in the low SNR region and 1 in the high SNR region. Note that the corresponding lower bounds $P_{1}=1-\exp{\left[\left((2^{\frac{R_{\mathrm{th}}}{T_{f}B}}-1\right)\lambda_{e}\gamma_{\mathrm{in}}\right)}^{\frac{\alpha }{2}}]$ and $P_{2}=1-\exp[-{\left(\left(2^{\frac{R_{\mathrm{th}}}{T_{f}B}}-1\right)\lambda_{e}/B_{\max}\right)}^{\frac{\alpha }{2}}]$ can be obtained from Lemmas 2 and 3, respectively. In Figure 12, we plot the outage probability curves versus the instantaneous SNR under the QoS constraint $\theta =10^{-4}$ , where the energy arrival rate is set to be 1, 2, and 3, respectively. As depicted in Figure 12, when energy arrival rate is 1, the battery-infinite outage probability is the same as battery-finite outage probability. When energy arrival rate is 2 or 3, the battery-infinite energy harvesting based wireless sensor networks achieve a smaller outage probability than the battery-finite energy harvesting based wireless sensor networks. This is because the optimal power control policy is not constrained by the battery capacity when energy arrival rate is 1. Thus, both battery-infinite and battery-finite energy harvesting based wireless sensor networks have the same outage probability. When energy arrival rate is 2 or 3, the optimal power control policy is limited by the battery capacity in battery-finite energy harvesting based wireless sensor networks. Thus, the battery-finite energy harvesting based wireless sensor networks have the larger outage probability than the battery-infinite energy harvesting based wireless sensor networks.

## 7. Conclusions

In this paper, we developed the statistical delay-bounded QoS-driven power control policies for energy harvesting based wireless sensor networks to maximize the effective energy efficiency. First, we analyzed the available energy constraints for the battery-infinite and battery-finite energy harvesting based wireless sensor networks, respectively. Then, we formulated the EEE maximization problems, solving which, we derived the optimal power control policies. Our analyses identified the key fact that, under various QoS constraints, the optimal power control policy for battery-infinite energy harvesting based wireless sensor networks varies between the E-WF scheme and E-CI scheme while the optimal power control policy for battery-finite energy harvesting based wireless sensor networks varies between the T-WF scheme and T-CI scheme. We also derived the threshold of the energy arrival rate to judge whether the EEE is limited by the battery capacity constraint or not. In addition, we analyzed the outage probabilities for energy harvesting based wireless sensor networks using our developed optimal power control policies. The obtained numerical results validated our analyses and showed that our developed QoS-driven power control policies can achieve the maximum EEE for energy harvesting based wireless sensor networks.
